# The Duality of Entropy/Extropy, and Completion of the Kullback Information Complex

**DOI:** 10.3390/e20080593

**Published:** 2018-08-09

**Authors:** Frank Lad, Giuseppe Sanfilippo, Gianna Agrò

**Affiliations:** 1 Department of Mathematics and Statistics, University of Canterbury, 8140 Christchurch, New Zealand; 2 Department of Mathematics and Computer Science, University of Palermo, 90123 Palermo, Italy; 3 Department of Economics, Business, and Statistics, University of Palermo, 90128 Palermo, Italy

**Keywords:** entropy, extropy, relative entropy/extropy, prevision, duality, Fermi–Dirac entropy, Kullback symmetric divergence, total logarithmic scoring rule, Pareto optimal exchange

## Abstract

The refinement axiom for entropy has been provocative in providing foundations of information theory, recognised as thoughtworthy in the writings of both Shannon and Jaynes. A resolution to their concerns has been provided recently by the discovery that the entropy measure of a probability distribution has a dual measure, a complementary companion designated as “extropy”. We report here the main results that identify this fact, specifying the dual equations and exhibiting some of their structure. The duality extends beyond a simple assessment of entropy, to the formulation of relative entropy and the Kullback symmetric distance between two forecasting distributions. This is defined by the sum of a pair of directed divergences. Examining the defining equation, we notice that this symmetric measure can be generated by two other explicable pairs of functions as well, neither of which is a Bregman divergence. The Kullback information complex is constituted by the symmetric measure of entropy/extropy along with one of each of these three function pairs. It is intimately related to the total logarithmic score of two distinct forecasting distributions for a quantity under consideration, this being a complete proper score. The information complex is isomorphic to the expectations that the two forecasting distributions assess for their achieved scores, each for its own score and for the score achieved by the other. Analysis of the scoring problem exposes a Pareto optimal exchange of the forecasters’ scores that both are willing to engage. Both would support its evaluation for assessing the relative quality of the information they provide regarding the observation of an unknown quantity of interest. We present our results without proofs, as these appear in source articles that are referenced. The focus here is on their content, unhindered. The mathematical syntax of probability we employ relies upon the operational subjective constructions of Bruno de Finetti.

## 1. Introduction

After some seventy years of extensive theoretical and applied research on the conception and application of entropy in myriad fields of science, informatics, and engineering, it may be surprising to find that there is another substantive dimension to the concept that has only recently been exposed. In a word, the *entropy* measure of disorder in a probability distribution is formally entwined with a complementary dual measure that we have designated as *extropy*. In assessing the probability of an event with only two possible outcomes, the entropy and extropy are identical. However, the measures bifurcate when the number of possibly observable values of a quantity is three or more, assessed with a probability mass function. As companions, these two measures relate as do the positive and negative images of a photographic film, and they contribute together to characterizing the information in a distribution in much the same way. In the present exposition, we shall merely report the main results discursively without proofs. Proofs appear in three published articles [1,2,3] which also supply relevant motivation, thoughtful discussion, and complete development in a matter of some sixty pages all told. Since the topics we cover are extensive, the summary provided here will necessarily be cryptic in places.

We begin by identifying the dual equations that entwine entropy/extropy as a bifurcating measure, and by displaying contours of iso-entropy and iso-extropy probability mass vectors (pmvs) within the triangular unit-simplex appropriate to a problem with three measurement possibilities. In this context, we also portray the alternative refinement axioms that support the duality. Furthermore, the notion of *relative entropy* was originally envisaged in order to provide for invariance of a continuous entropy measure with respect to monotonic transformations of the measurement scale. We show how the measurement of relative extropy completes this notion in a natural way. A unified understanding of the dual measures is provided within the larger scheme of general Bregman divergences.

An examination of Kullback’s symmetric distance measure between two pmvs then reveals that it can be generated equivalently by three distinct paired measures of them. It becomes evident that the computation of Kullback’s distance needs to be supplemented with computed values of all three generators in order to portray the information content of the two forecasting pmvs. The practical value of this four-dimensional measure is found in its relevance to the evaluation of alternative forecasts via the theory of proper scoring rules. Our results identify an isomorphism between the Kullback information complex so defined and the two forecasters’ expectations of their achieved total logarithmic score evaluations, each for its own and for the other’s.

The concluding exposition concerns the related discovery of a desirable method for evaluating the quality of alternative forecasts, which avoids an arbitrariness inherent in the mere comparison of achieved proper score evaluations. The consequences of the forecasters’ differing expectations of their scores supports a Pareto optimal exchange of their achieved scores that they both would embrace as a fair assessment of their comparative forecasting performances.

Our presentation proceeds in the context of the operational subjective theory of probability, deriving from the mathematical and foundational constructions of Bruno de Finetti in the twentieth century. Notable expositions appear in his publications [4,5]. The differences between this understanding and what is considered standard statistical practice are fundamental, even allowing for Bayesian procedures. Probability distributions are not considered to be unobservable generators of random data observations, requiring estimation. Instead, they are numerically codified assertions of uncertain scientists/engineers/designers regarding unknown but observable quantities, to be used in guiding practical decisions. Even the mathematical semantics of probability itself are recognizably different from standard usage. For example, an event is not characterised as a set but as a number, either zero or one, whose value is unknown to the proponent of a probability. Notationally, an event may be denoted by a parenthetic indicator expression such as $\left(X=x_{i}\right)$ when the quantity *X* has several possible values. In such a case, the parenthetic expression is meant to indicate one (1) if the interior expression is observed to be true, and zero (0) if false. Otherwise, the syntax is fairly natural. In some places, we shall defer to common practice, as in denoting an expectation operator by $E_{p}{(}^{.})$ rather than using prevision $P_{p}{(}^{.}),$ which would be natural to a subjectivist. This is an operator that unifies expectation and probability in de Finetti’s formulation, treating expectation as the primitive notion of uncertainty specification. A pedagogical introduction to operational subjective methods and their motivation can be found in the controversial text of Lad [6].

Inspiration for discovering the dual complementarity of entropy/extropy arose from our interest in the use of *proper scoring rules* for assessing the quality of alternative probability distributions asserted as forecasts of observable quantities of interest. These are introduced in the aforementioned text, having been formalised in the final technical contribution of Savage in [7]. From the subjectivist perspective, the use of proper scoring rules is meant to completely replace the entire construct of hypothesis testing. This is recognised as a meaningless endeavor of searching for non-existent probabilistic generators of supposedly random phenomena. An extensive literature review appeared in [8] with a slightly different emphasis. However, the duality of entropy/extropy is a formal mathematical property of the pair of functions. We expect it to be relevant to most all fields in which the concept of entropy has proved useful.

## 2. Results

Consider a measurable quantity *X* with a finite discrete realm of N possible instantiations, *R*$\left(X\right)=\left\{x_{1},x_{2},...,x_{N}\right\}$. Our analysis concerns the character of two different asserted probability mass vectors (pmvs) for these possible outcomes: **p**${}_{N}=\left(p_{1},\ldots ,p_{N}\right)$ and **q**${}_{N}=\left(q_{1},\ldots ,q_{N}\right)$, along with two other relevant pmvs to be denoted by **s**${}_{N}=\left(s_{1},\ldots ,s_{N}\right)$ and **t**${}_{N}=\left(t_{1},\ldots ,t_{N}\right)$. Each component of vectors such as **p**${}_{N}$ is a probability: $p_{i}=P\left(X=x_{i}\right),\;i=1,\ldots ,N$.

### 2.1. Complementary Duality

The well-known *entropy* measure of a pmv is specified by the function value $H\left(p_{N}\right)\;\equiv \;-\;\sum_{i=1}^{N}\;p_{i}\;\log\left(p_{i}\right)$. This measure has a complementary dual in the measure defined by $J\left(p_{N}\right)\;\equiv \;-\sum_{i=1}^{N}\left(1-p_{i}\right)\;\log\left(1-p_{i}\right)$, which we designate as *extropy*.

It should be evident that, when N=2, the entropy $H(p_{2})$ and extropy $J(p_{2})$ are identical. However, when N>2, the measure bifurcates to yield distinct paired measurements $\left[H\left(p_{N}\right),J\left(p_{N}\right)\right]$.

The dual equations relating these two measures are

$$J\left(p_{N}\right)\;\;=\;\;\sum_{i=1}^{N}\;H\left(p_{i},1-p_{i}\right)\;-\;H\left(p_{N}\right)$$

and symmetrically,

$$H\left(p_{N}\right)\;\;=\;\;\sum_{i=1}^{N}\;J\left(p_{i},1-p_{i}\right)\;-\;J\left(p_{N}\right).$$

Replacing the function names *H* by *J* in either of these equations while simultaneously replacing *J* by *H* yields the other equation. Formally, this is the source of the duality.

This equation pair arises from the feature that H(**p**${}_{N})+J($**p**${}_{N})\;=\;\;\sum_{i=1}^{N}\;H\left(p_{i},1-p_{i}\right)$. While the sum on the left has been known as Fermi–Dirac entropy, the duality of the summands has been long unrecognised. The dual does *not* constitute an *involution*, which would mean that the dual of a dual function constitutes the original function. Instead, extropy is a *complementary* dual of entropy. This arises from the fact that the extropy of a pmv **p**${}_{N}$ equals a linearly rescaled measure of entropy of its complementary pmv **s**${}_{N}\equiv {\left(N-1\right)}^{-1}\left(1_{N}-p_{N}\right)$. That is, $J\left(p_{N}\right)\;=\;\left(N-1\right)\;\left[H\left(s_{N}\right)-\log\left(N-1\right)\right]\;.\;$

The transformation of the pmv $p_{N}$ to its complement $s_{N}$ is a contraction.

**Figure 1:**
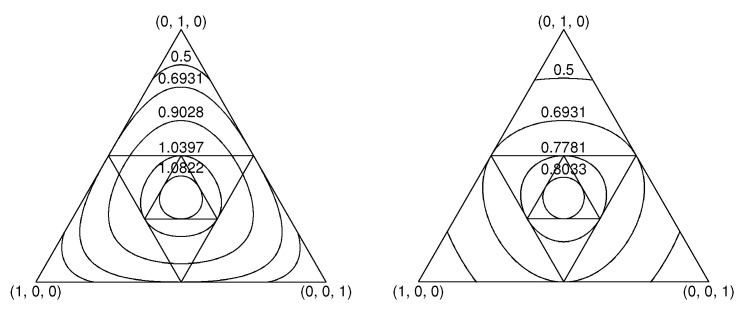
On the **left** are equal-entropy contours of distributions within the 2D unit-simplex, **S**${}^{2}$. On the **right** are equal-extropy contours of distributions. The inscribed equilateral triangles exhibit sequential contractions of the range of the complementary transformation from $p_{3}$ vectors to their complements $q_{3}$, and then in turn from these $q_{3}$ vectors to their complements, and so on. The fixed point of all contraction mappings is the uniform distribution (pmv).

A visual display of the dual functions $H{(}^{.})$ and $J{(}^{.})$ appears in Figure 1. It exhibits equal-entropy contours and equal-extropy contours for pmvs $p_{3}$ in the two-dimensional unit simplex.

### 2.2. Axiomatic Construction of Entropy and Extropy

Shannon had initially characterised the entropy function as the unique function that satisfies three axioms [9]. However, the third axiom puzzled him. It concerns the gain in entropy incurred when a component probability of a pmv is refined to specify probabilities for two constituents:

$$H\left(tp,\left(1-t\right)p,1-p\right)\;\;=\;\;H\left(p,1-p\right)\;+\;p\;H\left(t,1-t\right)\;\;\;\;\;\;\;\;\;\;\mathrm{for}\;\mathrm{any}\left(p,t\right)\in {\left(0,1\right)}^{2}\;.$$

Shannon recognised the usefulness of the theory this axiom supports, particularly in identifying the entropy in the joint pmv for a pair of quantities as $H\left(X,Y\right)\;=\;H\left(X\right)+P[H\left(Y|X=x^{o}\right)]$, where $x^{o}$ is the observed value of *X*. However, he expressed some concern that he could not provide salient motivation for it. Jaynes wondered aloud in his text ([10], p. 351) some years later whether the axiom might be proved somehow to be uniquely satisfactory in characterising a measure of information, or whether it could be sensibly replaced. Our realisation of the complementarity of extropy with entropy provides the alternative axiom supporting the dual measure for which Jaynes was searching:

$$J\left(tp,\left(1-t\right)p,1-p\right)\;\;=\;\;J\left(p,1-p\right)\;+\;\Delta \left(p,t\right)\;\;\;\;\;\;\;\;\;\;\;\;\;\mathrm{for}\;\mathrm{any}\left(p,t\right)\in {\left(0,1\right)}^{2}\;,$$

where Δ(p,t)= (1−p)log(1−p)−(1−tp)log(1−tp)−{1−(1−t)p}log{1−(1−t)p}.

**Figure 2:**
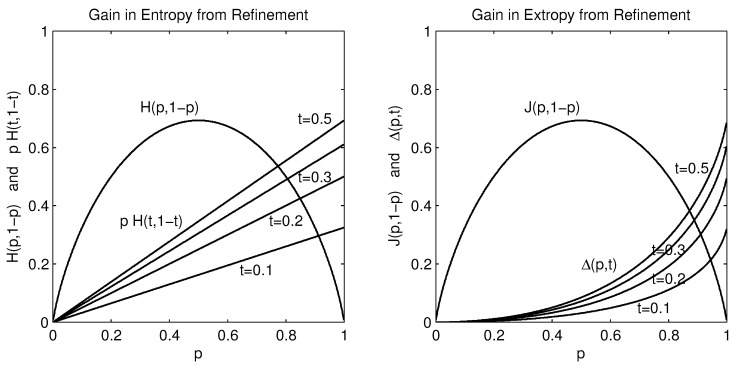
The entropies (at **left**) and extropies (at **right**) for refined distributions [tp,(1−t)p,(1−p)] equal the entropy/extropy for the base probabilities, H(p,1−p)=J(p,1−p), plus an additional component. This component is linear in *p* at the constant rate H(t,1−t) for entropy, and non-linear in *p* for extropy at a rate that increases with the size of *p*.

The algebraic detail is less important here than is the content. This is exhibited graphically in Figure 2. Whereas the entropy gain is linear in the size of the probability *p* that is refined, at a rate depending only on the entropy of the partitioning fraction *t*, the extropy gain also increases with the size of *p* but at a rate that increases with the size of the refined *p* as well. Details are best studied in [1].

### 2.3. Relative Entropy and Its Complementary Dual

Shannon’s development provided an intuitive suggestion for a representation of entropy as applied to a continuous density function. Recognised by Kolmogorov [11] as lacking invariance with respect to monotonic transformations of the variable quantity under consideration, the theory was completed by recourse to the Kullback divergence in the classic text [12]. In a discrete context, the entropy in a mass function $p_{N}$ relative to to another, $q_{N}$, was identified as the *relative* entropy function $D\left(p_{N}∥q_{N}\right)\;\equiv \;\sum_{i=1}^{N}\;p_{i}\;\log\left(\frac{p_{i}}{q_{i}}\right)$, which is also known as the Kullback divergence between $p_{N}$ and $q_{N}$. We have found that this measure too has a complementary dual that we identify as relative extropy: $D^{c}\left(p_{N}∥q_{N}\right)\equiv \sum_{i=1}^{N}\;\left(1-p_{i}\right)\log\left(\frac{1-p_{i}}{1-q_{i}}\right)$. The dual equations are found to be

$$\;D^{c}\left(p_{N}∥q_{N}\right)\;=\;\sum_{i=1}^{N}\;D\left[\left(p_{i},1-p_{i}\right)∥\left(q_{i},1-q_{i}\right)\right]-D\left(p_{N}∥q_{N}\right),$$

and symmetrically,

$$\;D\left(p_{N}∥q_{N}\right)\;=\;\sum_{i=1}^{N}\;D^{c}\left[\left(p_{i},1-p_{i}\right)∥\left(q_{i},1-q_{i}\right)\right]-D^{c}\left(p_{N}∥q_{N}\right).$$

The complementarity is seen in the result that

$$D^{c}\left(p_{N}∥q_{N}\right)\;=\;\left(N-1\right)\;D\left(s_{N}∥t_{N}\right),\;\;$$

where $s_{N}$ and $t_{N}$ are the pmvs complementary to $p_{N}$ and $q_{N}$. That is, **s**${}_{N}={\left(N-1\right)}^{-1}\left(1_{N}-p_{N}\right)$ and **t**${}_{N}={\left(N-1\right)}^{-1}\left(1_{N}-q_{N}\right)$. This result mimics the simple complementary duality of $J(p_{N})$ with $H(s_{N})$ for the complementary pmvs $p_{N}$ and $s_{N}$.

### 2.4. Unification via Bregman Divergences: The Continuous Situation

The structure of Bregman divergences both unifies our understanding of the (entropy, extropy) duality, and provides a basis for characterising the duality of these measures for density functions. We report here only some results, using standard notation. Readers unfamiliar with these divergences will find an introduction in [13]. In a discrete context, it is well known that the Kullback directed distance measure between two vectors, $D(p_{N}∥q_{N})$, is a Bregman divergence with respect to the separable Bregman function $\Phi \left(p_{N}\right)=-H\left(p_{N}\right)$. See [14,15] for example. This is commonly denoted by writing $d_{\Phi }\left(p_{N},q_{N}\right)=D\left(p_{N}∥q_{N}\right)\geq 0$, with equality applying if and only if $p_{N}=q_{N}$. The same is true of the complementary distance: $D^{c}\left(p_{N}∥q_{N}\right)$ is a Bregman divergence with respect to the separable Bregman function $\Phi^{c}\left(p_{N}\right)=-J\left(p_{N}\right)$. Specifically, $d_{\Phi^{c}}\left(p_{N},q_{N}\right)=D^{c}\left(p_{N}∥q_{N}\right)$, again non-negative. We shall also have reason to address the cross-entropy and cross-extropy functions: $CH\left(p_{N}∥p_{N}\right)\equiv \sum_{i=1}^{N}p_{i}\;\log\;q_{i}$, and $CJ\left(p_{N}∥p_{N}\right)\equiv \sum_{i=1}^{N}\left(1-p_{i}\right)\;\log\;\left(1-q_{i}\right)$.

In a continuous context, the relative entropy between two density functions *f* and *g* defined over an interval $\left[x_{1},x_{N}\right]$ can be identified as a Bregman divergence with respect either to the function ϕ(f)=flog(f) or ϕ(f)=flog(f)+(1−f). This is typically denoted by writing $d\left(f∥g\right)\;=\;B_{\phi }\left(f,g\right)=\;\int_{x_{1}}^{x_{N}}f\left(x\right)\log\left(\frac{f(x)}{g(x)}\right)dx$. We have found that its complementary dual specifies the relative extropy between *f* and *g* as a Bregman divergence as well, with respect either to the function $\theta \left(f\right)=\frac{1}{2}f^{2}$ or $\theta \left(f\right)=-f+\frac{1}{2}f^{2}$. In explicit notation, we write $d^{c}\left(f∥g\right)=B_{\theta }\left(f,g\right)=\frac{1}{2}\int_{x_{1}}^{x_{N}}{\left[f\left(x\right)-g\left(x\right)\right]}^{2}dx$. The Kullback divergence and one-half of the $L_{2}$ metric are thus recognised as complementary duals.

*N.B.* We shall presume in what follows that the size of all vectors is *N*, omitting it as a subscript in vector notation. For example, the divergence $D(p_{N}∥q_{N})$ will now be written more simply as D(p∥q).

### 2.5. The Kullback Information Complex

**Figure 3:**
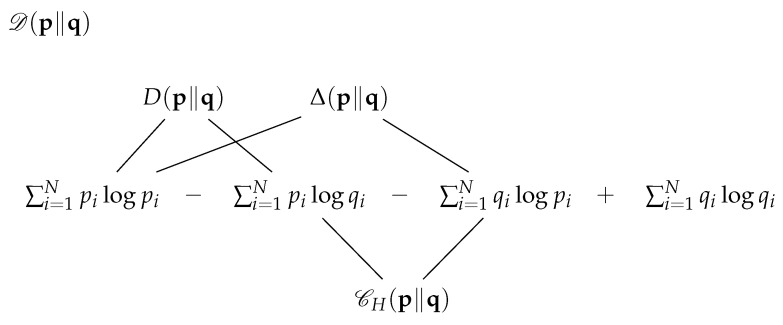
Schematic display of the symmetric Kullback divergence D(p∥q), which shows that it can be generated equivalently by three distinct pairs of summands. These are specified by the directed divergence D(p∥q), by an alternative difference Δ(p∥q), and by the cross-entropy sum $C_{H}(p∥q)$.

Since the Kullback divergence D(p∥q) is not symmetric, a symmetric distance (which still does not satisfies the triangle inequality property) is defined by a sum of the two directed divergences (see [12,16]): D(p∥q)≡D(p∥q)+D(q∥p). However, two alternative generations of the symmetric divergences can be recognised by viewing Figure 3.

Algebraically, this scheme can be described by the three equivalent equations:

(1) $$\begin{array}{ccccc} D(p∥q) & \equiv & \;D(p∥q) & + & \;\;D(q∥p)\\ & = & \;\Delta (p∥q) & + & \;\;\Delta (q∥p)\\ & = & \;C_{H}(p∥q)\; & - & \;\frac{1}{2}\;\left[C_{H}(p∥p\right)+C_{H}(q∥q)]\geq \;\;0,\;\;\;\;\; \end{array}$$

where $\Delta \left(p∥q\right)\equiv \;\sum_{i=1}^{N}\;\left(p_{i}-q_{i}\right)\;\log\left(p_{i}\right)$, and $C_{H}(p∥q)\equiv CH\left(p∥q\right)+CH\left(q∥p\right)$.

We observe that D(p∥q)=0 if and only if p=q. While the directed divergence $D{(}^{.}{∥}^{.})$ is non-symmetric, it specifies a self-divergence of zero for D(p∥p) and D(q∥q). The same is true of the directed measure $\Delta {(}^{.}{∥}^{.})$. In contrast, the cross-entropy sum $C_{H}\left(p∥q\right)\equiv CH\left(p∥q\right)+CH\left(q∥p\right)$ is already symmetric: $C_{H}\left(p∥q\right)=C_{H}\left(q∥p\right)$, while the self measures $C_{H}\left(p∥p\right)$ and $C_{H}\left(q∥q\right)$ are non-zero. These equal 2H(p) and 2H(q), respectively.

We can build on the awareness that these equations provide by defining a vectorial complex of information measures that supplement the symmetric divergence. Motivation will be found in its relation to four fundamental previsions that are relevant to the evaluation of a total logarithmic score, and in concerns cited in [17]. To begin, we define the *Kullback information complex* for the pmvs (p,q) as the vector $\left[D\left(p∥q\right),\;D\left(p∥q\right),\;\Delta \left(p∥q\right),\;C_{H}\left(p∥q\right)\right]$. Correspondingly, we define the *complementary Kullback information complex* as the vector $\left[D^{c}\left(p∥q\right),\;D^{c}\left(p∥q\right),\;\Delta^{c}\left(p∥q\right),\;C_{J}\left(p∥q\right)\right]$ on the basis of complementary functions that we should now expect:

$$\begin{array}{ccc} D^{c}\left(p∥q\right)\; & \equiv & \;D^{c}\left(p∥q\right)\;+\;D^{c}\left(q∥p\right),\\ \Delta^{c}\left(p∥q\right)\; & \equiv & \;\sum_{i=1}^{N}\;\left(1-p_{i}\right)\;\log\left(1-p_{i}\right)-\sum_{i=1}^{N}\;\left(1-q_{i}\right)\;\log\left(1-p_{i}\right)\;,\;\;\mathrm{and}\\ CJ(p∥q)\; & \equiv & \;-\sum_{i=1}^{N}\;\left(1-p_{i}\right)\;\log\left(1-q_{i}\right)\;,\;\;\mathrm{along}\;\mathrm{with}\\ C_{J}\left(p∥q\right)\; & \equiv & \;CJ(p∥q)+CJ(q∥p). \end{array}$$

The function CJ(p∥q) denotes cross-extropy. Using these functions, a display replicating Figure 3 can be produced for the complementary symmetric divergence $D^{c}\left(p∥q\right)$. In each instance where the probability $p_{i}$ or $q_{i}$ appears, it would be replaced by its complement, $1-p_{i}$ or $1-q_{i}$. See [2]. The functions $D^{c}\left(p∥q\right)$, $\Delta^{c}\left(p∥q\right)$, and the sum $C_{H}\left(p∥q\right)$ would each designate once again two selected summands of $D^{c}\left(p∥q\right)$. This would substantiate three parallel generating functions of $D^{c}\left(p∥q\right)$ appropriate to duality.

Finally, by summing the Kullback information complex and its complement, we obtain the *total Kullback information complex*:

$$[D\left(p∥q\right)+D^{c}\left(p∥q\right),\;\;\;D\left(p∥q\right)+\;D^{c}\left(p∥q\right),\;\;\;\Delta \left(p∥q\right)+\Delta^{c}\left(p∥q\right),\;\;\;C_{H}\left(p∥q\right)+C_{J}\left(p∥q\right)]\;.$$

We are now prepared to identify the contribution of entropy/extropy measures for the use of proper scoring rules for evaluating the comparative quality of alternative forecasting distributions.

### 2.6. Connections with Proper Scoring Rules: The Total Logarithmic Score

The operational subjective theory of probability supports the comparative evaluation of alternative probabilistic forecasts of measurable quantities via proper scoring rules. The *total logarithmic score* for p (denoted by $S_{TL}$) on the basis of the observation of *X* is specified as

$$S_{TL}\left(p,X\right)\;\;\equiv \;\;\sum_{i=1}^{N}\left(X=x_{i}\right)\;\log\left(p_{i}\right)\;+\;\sum_{i=1}^{N}\left(X\neq x_{i}\right)\;\log\left(1-p_{i}\right)\;.$$

Here, the parenthetic expressions surrounding equations denote the indicator 1 if the equation is observed to be true, and 0 if it is false. Thus, when *X* is observed to equal $x^{o}$, say, the first summand reduces to $log[P\left(X=x^{o}\right)]$ because all the other parenthetic expressions $\left(X=x_{i}\right)$ indicate a value of zero (0). The second summand then reduces to the sum of the logs for all other probabilities assessed for events that indicate $\left(X\neq x_{i}\right)$ for all the other measurement possibilities. Thus, when *X* is observed to equal $x^{o}$, then $S_{TL}\left(p,X\right)=\log p^{o}+\sum_{i=1}^{N}\;log\left(1-p_{i}\right)-\log\left(1-p^{o}\right)$, where $p^{o}=P\left(X=x^{o}\right)$. This scoring rule provides a complete score of a pmv, complete in the sense that *every* asserted component of this pmv is involved in determining the score. No assertion component avoids assessment. The observation of $X=x^{o}$ is relevant to each of them. When two alternative forecasting distributions are asserted via the pmvs p and q, we are interested in an assessment of their quality provided by four fundamental previsions: the two forecasters’ expectations of their total scores, each for one’s own score and for the score to be achieved by the other:

$$\begin{array}{ccc} E_{p}\left[S_{TL}\left(p,X\right)\right] & =\;\;\sum_{i=1}^{N}p_{i}\;\log\left(p_{i}\right)+\sum_{i=1}^{N}\left(1-p_{i}\right)\;\log\left(1-p_{i}\right) & =\;\;-[H(p)+J(p)]\;,\;\;\;\;\mathrm{and}\\ E_{p}\left[S_{TL}\left(q,X\right)\right] & =\;\;\sum_{i=1}^{N}p_{i}\;\log\left(q_{i}\right)+\sum_{i=1}^{N}\left(1-p_{i}\right)\;\log\left(1-q_{i}\right) & =\;\;-[CH(p∥q)+CJ(p∥q)]\;,\\ E_{q}\left[S_{TL}\left(q,X\right)\right] & =\;\;\sum_{i=1}^{N}q_{i}\;\log\left(q_{i}\right)+\sum_{i=1}^{N}\left(1-q_{i}\right)\;\log\left(1-q_{i}\right) & =\;\;-[H(q)+J(q)]\;,\;\;\;\;\;\mathrm{and}\\ E_{q}\left[S_{TL}\left(p,X\right)\right] & =\;\;\sum_{i=1}^{N}q_{i}\;\log\left(p_{i}\right)+\sum_{i=1}^{N}\left(1-q_{i}\right)\;\log\left(1-p_{i}\right) & =\;\;-[CH(q∥p)+CJ(q∥p)]\;. \end{array}$$

### 2.7. The Isomorphism of the Total Kullback Complex with Four Fundamental Previsions

Each of the four components of a total Kullback complex is a different linear combination of the various entropies, extropies, cross entropies and cross extropies that constitute these four fundamental previsions. These combinations happen to be ordered in such a way that the two four-dimensional vector functions are isomorphic, related by the linear equations

$$\left[\begin{array}{c} D\left(p∥q\right)+D^{c}\left(p∥q\right)\\ D\left(p∥q\right)+D^{c}\left(p∥q\right)\\ \Delta \left(p∥q\right)+\Delta^{c}\left(p∥q\right)\\ C_{H}\left(p∥q\right)+C_{J}\left(p∥q\right) \end{array}\right]=\left[\begin{array}{cccc} 1 & -1 & 1 & -1\\ 1 & -1 & 0 & \;\;0\\ 1 & \;\;0 & 0 & -1\\ 0 & -1 & 0 & -1 \end{array}\right]\times \left[\begin{array}{c} E_{p}\left[S\left(X,p\right)\right]\\ E_{p}\left[S\left(X,q\right)\right]\\ E_{q}\left[S\left(X,q\right)\right]\\ E_{q}\left[S\left(X,p\right)\right] \end{array}\right].$$

The inverse transformation is

$$\left[\begin{array}{c} E_{p}\left[S\left(X,p\right)\right]\\ E_{p}\left[S\left(X,q\right)\right]\\ E_{q}\left[S\left(X,q\right)\right]\\ E_{q}\left[S\left(X,p\right)\right] \end{array}\right]=\left[\begin{array}{cccc} 0 & \;\;0.5 & \;\;0.5 & -0.5\\ 0 & -0.5 & \;\;0.5 & -0.5\\ 1 & -0.5 & -0.5 & -0.5\\ 0 & \;\;0.5 & -0.5 & -0.5 \end{array}\right]\times \left[\begin{array}{c} D\left(p∥q\right)+D^{c}\left(p∥q\right)\\ D\left(p∥q\right)+D^{c}\left(p∥q\right)\\ \Delta \left(p∥q\right)+\Delta^{c}\left(p∥q\right)\\ C_{H}\left(p∥q\right)+C_{J}\left(p∥q\right) \end{array}\right].\;$$

Each of the three directed distance function pairs composing the total Kullback complex measures a distinct information source for understanding the full content of Kullback’s symmetric distance. The symmetric distance measure is incomplete on its own: it needs to be supplemented by three more components if it is to represent the information content of the pmv assertions p and q. On its own, the symmetric distance measure confounds distinct information characteristics in the summed cross-entropies/extropies less the summed own-entropies/extropies of the two distributions. The three companion generating measures in the complex allow the dissection of this amalgam in a way that illuminates the contributions of each forecasting distribution to its composition. The linear relation of the complex to the four fundamental previsions exposes the meaningful content of the dual measure.

### 2.8. Pareto Optimal Exchange of Achieved Proper Scores

Long honoured empirical scoring of comparative forecast distributions on the basis of computed proper scores suffers from a challenging puzzle. We can resolve it in a novel way. For purposes of discussion here, we shall consider again the context provided by the Total Log scoring rule, supposing the forecaster *p* asserts a pmv p for the observable *X*, while forecaster *q* asserts q. While the language of “forecasting” may suggest weather forecasts or forecasts of economic indicators, the sense of the theory is applicable to any type of unknown measurement whatever. For examples, the theory of quantum mechanics specifies probabilistic forecasts of the experimental polarization behaviour (reflection or absorption) of a photon when engaging a polarizing material at a various angles; and the theory of genetics specifies probabilistic forecasts regarding the corpulent status of a living organism that embodies a particular genetic code.

No assertion of a probabilistic forecasting distribution can be considered to be “wrong”. For when one asserts a probability distribution over possible measurement values of a quantity, one is merely expressing one’s uncertain opinions in a precise prescribed fashion. There is nothing wrong about being uncertain. Nonetheless, it is useful for many reasons to gauge the quality of a forecaster’s probability assertions in terms of the quantity actually observed.

A proper scoring function is designed both to promote honesty and accuracy in the assessment of one’s personal probability distribution, and also to allow an evaluation of the quality of the forecast in light of the observation that is eventually made. The scoring function for the pmv p on the basis of the observation of *X* is denoted by S(p,X). The person *p* who asserts p is uncertain both about the value of *X* itself and also about the score that will be obtained when *X* is observed. The scoring rule is said to be *proper* if *p*’s expected value of the score to be awarded to p exceeds *p*’s expectation of the score to be awarded to any other pmv. There can be no gain expected by strategically proffering as one’s own a pmv different from the probabilities to which one actually subscribes. There are many functions that qualify as proper scoring functions. Each one of them is associated with a different manner of utility valuation for experiencing the various possible values of *X* while assessing their probabilities as p. The total logarithmic score which we have been entertaining heretofore is one of them that has many desirable properties. The enigma we shall now address would pertain to any of them, however. It pertains to the use of such a scoring rule in comparing the quality of different forecasting distributions. How should we compare the relative qualities of different probabilistic forecasts on the basis of accumulating proper scores of their pronouncements regarding a sequence of data observations?

In current applications, the routine award of the score S(p,X) to forecaster *p* for comparison with an award of S(q,X) to forecaster *q* begs an interesting question. The assertion of p, for example, implies an indifference to a Net Gain score characterised by $NG\left(p,X\right)\equiv S\left(p,X\right)-E_{p}\left[S\left(p,X\right)\right]$. Clearly $E_{p}\left[NG\left(p,X\right)\right]=0$. From *p*’s point of view, it would be an arbitrary determination to be awarded S(p,X) or to be awarded $E_{p}\left[S\left(p,X\right)\right]$ for comparison with an award to forecaster *q*, for both of these are valued identically by *p*. The same consideration would apply to forecaster *q* who would regard an award of S(q,X) as opposed to $E_{q}\left[S\left(q,X\right)\right]$ as arbitrary, for *q* assesses each of these with the same expectation. Should these two forecasters trust a comparison of their expertise on the basis of their accumulated proper score values?

Our answer is “No!”. The novel resolution we propose to this enigma arises from considering each of the forecasters’ expectations of the scores to be achieved by the other, for, in contrast, forecaster *p* does *not* assert an expectation of the Net Gain score to be achieved by *q* as equal to 0, but rather some number either greater or smaller: $E_{p}\left[NG\left(q,X\right)\right]\neq 0$. Thus, *p* would be happy to trade the value of NG(p,X) with *q* in return for NG(q,X), either its positive or negative value according to the sign of $E_{p}\left[NG\left(q,X\right)\right]$, should such an exchange be on offer. As is the case, forecaster *q* would also be eager to offer NG(q,X) to *p* in return for NG(p,X) (again either its positive or negative value, appropriately). Both parties would be happy to make such an exchange of their Net Gain scores, as both of them expect to make a positive gain from such a trade. In economic lore, such an exchange between two parties is said to be a “Pareto optimal exchange”. In other types of exchanges, either or both of the traders may assess their utilities as diminished by a trade. This Pareto exchange would allow a comparative evaluation of the quality of the two forecasting distributions that both *p* and *q* would be happy to engage. Rather than accumulating the values of their own raw proper scores on the basis of the observation sequence, we have identified that the appropriate accumulation for each is the net gain that is achieved by the other! Neither of them would consider there to be anything arbitrary about it. A final qualification is that a scaling of the two sides of the exchange could ensure that both forecasters would assess the net gain offered with the same variance as the net gain received in return. Details appear in (Section 3, [2]).

**Figure 4:**
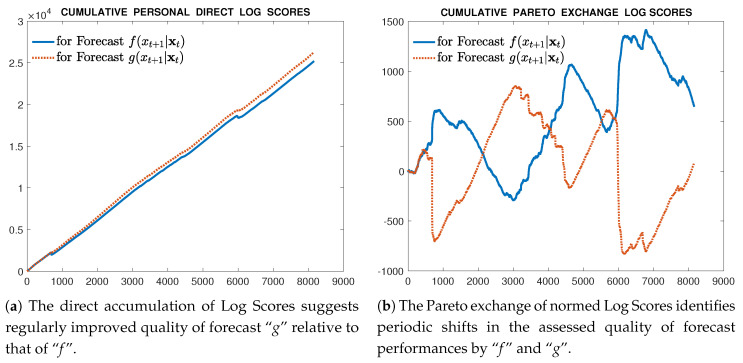
Comparative results of accumulating Direct Scores and Pareto exchanged Scores for the same two forecasting distributions and data sequence.

We have not yet completed the analysis of a full application. However, we can provide here an alluring glimpse of a partial graphical result in Figure 4. This displays how different an assessment of two sequential forecast densities can be when based upon their accumulating direct proper scores and when based upon their Pareto exchanged scores. Details of the data and the theoretical scientific issues involved must await a complete report. On the left side of the figure are portrayed the simple accumulating logarithmic scores of two sequential forecasting densities over a data sequence of some 8000 observations. The one labeled Forecast $f(x_{t+1}|x_{t})$ is a mixture-Gaussian distribution sequence. The one labeled Forecast $g(x_{t+1}|x_{t})$ is a mixture-Exponential-power distribution, designed to exhibit fat tails relative to the mixture-Gaussian. The fairly regular gain observed in the accumulating score of the forecasting scheme $g{(}^{.}{|}^{.})$ relative to $f{(}^{.}{|}^{.})$ seems to support a conclusion that $g{(}^{.}{|}^{.})$ provides a more accurately informative forecast. In contrast, on the right side, the comparative accumulating Pareto exchanged log scores for the same two sequences of forecasting distributions appear. The results are strikingly different. Not only do these accumulated exchange scores favour the mixture $f(x_{t+1}|x_{t})$ over the fat-tailed density $g(x_{t+1}|x_{t})$ by the end of the data series, but the sequential scoring identifies regular changes of fortune in the assessments of the two forecasting schemes throughout the study period. This is the full extent of results we are able to display at the moment, but it motivates us to conclude at least that examination of the Pareto exchange of scores is meritorious.

## 3. Conclusions

This presentation has been designed to promote recognition of the duality of a paired measure of probability distributions, entropy/extropy. Publications cited herein provide extensive discussion, motivation, and proofs of the results we have mentioned. It is hoped that readers involved in the many applications of entropy to the assessment of uncertainty may be intrigued to consider the relevance of extropy to their deliberations. One such application to methods of automatic speech recognition has already appeared in [18]. Several implications for the analysis of order statistics have been discussed in [19,20,21].

References
